# Strengthening adult mosquito surveillance in Africa for disease control: learning from the present

**DOI:** 10.1016/j.cois.2023.101110

**Published:** 2023-12

**Authors:** Zanakoungo I Coulibaly, Steve Gowelo, Issouf Traore, Rex B Mbewe, Willy Ngulube, Evelyn A Olanga, Adilson J DePina, Antoine Sanou, Sylvester Coleman, Julie-Anne A Tangena

**Affiliations:** 1 Institut Pasteur de Côte d’Ivoire, Cote d’Ivoire; 2 Malaria Alert centre of the Kamuzu University of Health Sciences, Malawi; 3 University of California San Francisco, Malaria Elimination Initiative, USA; 4 Malawi University of Business and Applied Sciences, Malawi; 5 Malawi Liverpool Wellcome Trust, Malawi; 6 The National Malaria Elimination Centre, Zambia; 7 Malaria Elimination Program, Ministry of Health, Cabo Verde; 8 Centre National de Recherche et de Formation sur le Paludisme, Burkina Faso; 9 Institut Supérieur de Développement Durable, Université de Fada N’Gourma, Burkina Faso; 10 Vector Biology department, Liverpool School of Tropical Medicine, United Kingdom

## Abstract

Mosquito surveillance is essential to successfully control and eliminate mosquito-borne diseases. Yet, it is often done by numerous organizations with little collaboration, incomplete understanding of existing gaps, and limited long-term vision. There is a clear disconnect between entomological and epidemiological indices, with entomological data informing control efforts inadequately. Here, we discuss current mosquito surveillance practises across the heterogeneous disease landscape in Africa. We advocate for the development of mosquito surveillance strategic plans to increase the impact and functionality of mosquito surveillance. We urge for a proactive approach to set up centralized mosquito data systems under the custodian of national governments, focus on epidemiologically relevant mosquito data, and increase the robustness of mosquito surveillance using a more spatially explicit sampling design.

## Highlights

• Weak mosquito surveillance restricts effective disease control.
• We advocate for the development of mosquito surveillance strategic plans.
• Centralized mosquito data systems are essential for data-informed decision-making.
• More integration of entomological and epidemiological data is necessary.
• Technological advancements can improve the robustness of mosquito surveillance.

**Current Opinion in Insect Science** 2023, **60**:101110

This review comes from a themed issue on **Vectors and medical and veterinary entomology**

Edited by **Zainulabeuddin Syed**

For complete overview about the section, refer “Vectors and medical and veterinary entomology (2023)”

https://doi.org/10.1016/j.cois.2023.101110

2214–5745/© 2023 The Authors. Published by Elsevier Inc. This is an open access article under the CC BY license (http://creativecommons.org/licenses/by/4.0/).

## Introduction

Since the discovery of mosquitoes as a vector for human diseases at the end of the 19th century, mosquito surveillance has been an essential part of disease control [1]. Mosquito surveillance is the routine collection of mosquito data to document disease vectors, including their species composition, distribution, habitats, behavior, infection status, and insecticide susceptibility status. They are key to developing vector control strategies, which prevent disease transmission by exploiting mosquito biology. Vector control has one of the highest economic return rates in public health and is an essential part of mosquito-borne disease control 2, 3••. The deployment of insecticide-treated bed nets and indoor residual spraying has contributed significantly to the decrease in malaria cases [4]. However, the gains have plateaued with current vector control tools insufficient to advance control and eradicate malaria. Surveillance is crucial to adapt guidance to local dynamics [3]. It is also essential to understand emerging threats (such as the invasion of *Anopheles stephensi* from South Asia to East Africa and the increasing number of arboviral disease outbreaks 5, 6. There is no ground for complacency, with a continued need to investigate the ever-changing mosquito dynamics.

**Figure 1:**
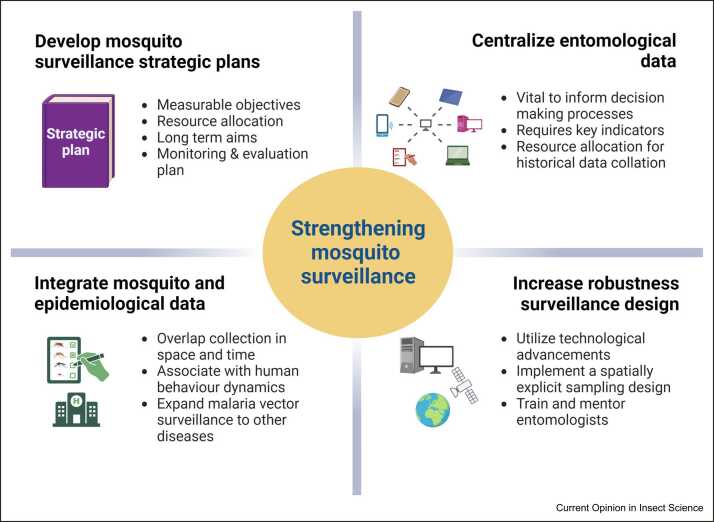
Summary of the main concepts to strengthen adult mosquito surveillance in Africa for disease control. Created with BioRender.com.

Weak surveillance systems restrict data-informed decision-making for the control and prevention of mosquito-borne diseases. The World Health Organization (WHO) has underlined the importance of mosquito surveillance in its Global Technical Strategy 2016– 2030 to retain gains and eliminate mosquito-borne diseases in the future, including its importance in monitoring insecticide resistance [7]. Mosquito surveillance is an expensive and time-consuming activity that requires highly skilled individuals with uncertain data output (i.e. not knowing if disease vectors will be collected). It is often dependent on external funding, with most African countries allocating their limited resources to disease treatment and control [5]. This has resulted in severe budgetary constraints and subsequently, haphazard implementation of mosquito surveillance activities. Most malaria vector control programs in sub-Saharan African countries struggle with inadequate resources, leading to data that are uninformative and sometimes misrepresentative. Funding limitations have led to the closure and temporary suspension of regular surveillance (sentinel) sites in countries such as Burkina Faso, Zambia, and Côte d’Ivoire 8, 9, 10, 11. It is more important than ever to have access to robust surveillance data to inform control efforts and expand surveillance toother disease vectors. It is crucial to share and compare mosquito surveillance practises in-country and across the continent. In the following, we draw on studies and personal experiences to discuss current mosquito surveillance activities across Africa and share our strategies to improve in the future (Figure 1).

## Develop mosquito surveillance strategic plans

Surveillance is often conducted with limited impact on the decision-making process. Disease control strategic plans do not include clear frameworks and pipelines of how mosquito surveillance data can be translated into policy, decisions, and actions, thus limiting data- driven policy 12, 13••, 14. We advocate for the development of mosquito surveillance strategic plans, which provide a framework for stakeholders to recognize areas of focus and long-term aims, with a monitoring and evaluation component to ensure limited resources are used appropriately. Countries such as Zambia are already considering this, with current surveillance strategy limited to the National Insecticide Resistance Monitoring and Management Plan that contains no impact indices for review, such as number of staff members trained or a list of essential mosquito surveillance indices to collect at each sentinel site [15]. In Burkina Faso, mosquito surveillance has been successfully integrated into vector control activities with the government presenting the surveillance as a package with vector control implementation, so that funding for surveillance does not need to be applied for separately. This integration is safeguarding clear activity pipelines and data-driven policy, thereby facilitating effective surveillance on some sentinel sites. It is crucial to also develop mosquito surveillance strategies for malaria-eradicated countries, such as Cabo Verde, where emphasis is on identifying early warning signs of re-emergence using surveillance at ports of entry and other high-risk areas.

We propose that mosquito surveillance strategic plans should consist of (at least) the objectives, a summary of available data, pipelines for activities, impact indices, and long-term aims. The WHO highlights five mosquito surveillance indicators (adult vector composition, vector behavior, adult vector resistance to insecticides, immature vector aquatic habitats, and proxies for transmission) [16]. These indicators can be used with the Entomological Surveillance Planning Tool and other guidance documents to identify gaps in protection and shortfalls of current interventions 17, 18. We recognize that at least vector species composition at high disease risk areas, preferred time and location for host seeking (biting), resistance to insecticides, and transmission potential should be included as a minimum standard of surveillance. The full list of key surveillance indicators, however, will differ per country or even per region, as different surveillance objectives require different indicators. For example, in an area where vector control is implemented, measuring insecticide resistance will be of great importance to ensure control efforts remain appropriate (i.e. species identification, susceptibility to insecticides, and biting and resting patterns). On the other hand, in an area where successful vector control (i.e. no insecticide resistance) is not resulting in a decrease in disease cases, focus might shift to the identification of putative vectors and their behavior in relation to the human population (i.e. identification of putative vectors, blood meal analysis, pathogen presence analysis, and vector host seeking behavior). This also means that there is no ideal number of sentinel sites or geographic coverage to recommend for the surveillance strategic plan. This is dependent on the objectives of the country and the heterogeneous landscape of the mosquito-borne diseases. However, we should expect vector surveillance programs to at least cover high disease incidence areas and areas where vector control efforts are implemented.

The strategic plan should have a monitoring and evaluation component with measurable outcomes. In Zambia, a minimum set of indicators have been selected for malaria programs with clear outcomes for 26 of their 51 sentinel sites, which are being reviewed annually. Initial evaluation showed less than 75% of planned activities were implemented due to limited resources, with the prioritization of treatment and control. The gap in resources led to a lack of entomological impact indicators, baseline data, and clear targets. An entomological Data Management Committee has now been established to address these concerns [11]. Similar monitoring systems have been established in Ghana [19] and are also being developed in Burkina Faso.

The strategic plan should address the structural challenge of resources and ensure they are used most adequately by prioritizing activities such as training medical entomologists. Most national malaria control programs (NMCP) lack enough entomological staff, sometimes requiring a doubling in staff, with constrained capabilities and insufficient training resources 12, 13••, 20, 21••. This lack of capacity and capabilities is especially evident at subnational level [22]. These challenges are further intensified for other mosquito-borne disease surveillance [23]. It is evident that strengthening malaria vector surveillance directly contributes to other mosquito monitoring efforts and conversely weakened malaria vector surveillance results in weak or nonexistent surveillance of other vector-borne diseases.

The strategic plan should include plans to develop, nurture, finance, and/or support medical entomology training in collaboration with universities and other academic organizations. We advocate for better retainment of talented and highly experienced entomologists. We encourage building the subnational entomological capacity and capabilities, which is greatly underfunded and underdeveloped. In Burkina Faso, local vector control teams are being introduced. Members in the team include Technician ingénieur de Génie sanitaire (Senior Technicians of Sanitary genius) and Ingénieur du Génie Sanitaire (Engineer in Sanitary genius), who have been trained on medical entomology. They are retrained annually and funding has recently been expanded to utilize the team for mosquito surveillance purposes also. Similarly, Kenya is investing in subnational entomologists, with the employment of county entomologists in each county as part of the County Health structure.

Presenting the strategic plan will help inform stakeholders on focus areas and help streamline data collection, analysis, and presentation across the many organizations involved in surveillance activities. To encourage participation and cooperation, the strategic plan should be written in strong collaboration with stakeholders. National organizations can be reached using platforms such as a vector control technical working group, which are key for knowledge translation and evidence generation [22]. They consist of representatives from at least academia, nongovernmental organizations, national government bodies, and vector control implementation groups. The establishment of these groups has improved knowledge sharing, data integration, and mosquito surveillance. At present, at least Burkina Faso, Cameroon, Côte d’Ivoire, Ghana, Zambia, Kenya, and Malawi have established these platforms. Community stakeholders should also be engaged [20]. Community health volunteers, who are actively involved in mosquito surveillance activities using indoor light traps indoors, can represent communities and provide input, as has been exemplified in western Kenya [24]. The establishment of these volunteer networks is also being explored in Malawi and western Ghana 25, 26. Communication between countries and regions is also important, with organizations such as Pan-African Mosquito Control Association (PAMCA) providing important platforms for sharing knowledge and improving mosquito surveillance across Africa.

## Centralize entomological data

Comprehensive, complete, and timely mosquito surveillance data are essential for informed decision-making in mosquito-borne disease control. Yet, data sharing is presently limited, patchy, and slow. Mosquito surveillance is highly fragmented, with activities designed, implemented, and monitored by a diverse group of stakeholders: from governmental organizations, nongovernmental organizations, and academics to industry. For example, in Zambia, 51 mosquito sentinel sites are surveyed at different frequencies by at least five organizations (excluding academic and industry) [15]. Coordinating mosquito surveillance is thus challenging for NMCPs. They have insufficient resources for a centralized data storage and management system, including tools and capacity to process, analyze, and interpret data 13••, 27. Currently, the most common methods for storing data are still paper- and Excel- based [12], which greatly limits preservation and accessibility.

It is essential to establish a centralized database. This requires prioritization from all stakeholders and allocation of sustained funding and resources. Government organizations should be custodians of the centralized mosquito surveillance data and should lead the discussion on the minimum key indicators to be collected by all stakeholders in the future (as discussed in “Develop mosquito surveillance strategic plans”). These key indicators should be shared through the strategic plan, ensuring all organizations implementing surveillance use these indicators in the field. We believe that the centralized data set should include at least the date of collection, method of collection, collection time, collection location (Global Positioning System (GPS) and indoor/outdoor), identification of species, method of identification, sex of mosquito, and organization/person responsible for data. Depending on the objectives highlighted in the strategic plan, this list should be expanded to include information on other indicators such as resistance status to specific insecticides, parity, abdominal status, and blood meal source. Discussions should be held about the workflow for genomic data sharing specifically, including its ownership and how to store these large data sets, as future surveillance tools will become more dependent on genetic analysis. Centralization of historical data will be more challenging, as organizations have different collection forms, storage methods, data sharing policies, and limited resources to collate the data. This process is time-consuming and labor-intensive, requiring significant investment. The key for its success will be to identify sufficient resources to support a team of data scientists and entomologists to pro-actively collect and merge the databases into a centralized system. The long-term goal is for NMCP to collate, analyze, and visualize all mosquito surveillance data, relate this to centrally available epidemiological data, so that it can be shared with policymakers in real time to inform decision-making processes. It should become standard practice for all mosquito surveillance projects to share their data through this centralized system in real time, with clear agreements on access restrictions in line with national and international agreements such as the Nagoya protocol. Data should also be accessible for local stakeholders, to ensure they have ownership and understanding of their local dynamics with flexibility to adjust to changing local needs.

Many central storage systems are available. For example, in Malawi, Research Electronic Data Capture (REDCap) is used, while Ghana, Zambia, and Kenya are adopting the District Health Information Software (DHIS2) Entomology database. There are also free programs available such as epicollect5 [28]. Unfortunately, full centralization of entomological data remains rare, especially at country level. The insecticide resistance (IR) mapper (https://www.irmapper.com/) and the WHO’s Malaria threat maps (https://apps.who.int/malaria/maps/threats) have shown the value of consolidating mosquito surveillance data, specifically insecticide resistance reports. These have been highly cited and shared. There are other important initiatives such as Vector Atlas (https://vectoratlas.icipe.org/) and the Global Vector Hub (https://globalvectorhub.lshtm.ac.uk/), which are working on improving access to mosquito surveillance data on a global scale. Investment in technology and personnel is necessary to establish a centralized database with a strong infrastructure, including technical equipment, backup systems, and training in data cleaning and management. To further support centralization, mosquito surveillance protocols should be openly accessible to support standardization of surveillance designs, decrease workload for entomologists, and simplify comparison of data across studies, locations, and time. The use of standardized data collection forms will further help accelerate data collection and availability [29].

## Collect epidemiologically relevant mosquito data

Presently, mosquito surveillance is narrowly focussed on the collection of primary malaria vector species and their anticipated biting behavior. This information is important to understand the impact of control efforts and has supported the wide decline of malaria across the continent. However, a disconnect between entomological and epidemiological indices remains. Traditionally, epidemiological data collection is centered around health clinics and their catchment areas, while vector data are collected with emphasis on historic collection activities instead of relevance to disease incidence. It has therefore been difficult to evaluate the impact of local interventions when clinical and entomological data are on a different spatial and temporal scale. Examples from Malawi and Kenya show the challenges of collecting malaria-infective mosquitoes and relating their dynamics to malaria incidence 30, 31, 32. Existing surveillance practices may not always effectively estimate disease indicators, capture data for other mosquito-borne diseases, or fully understand residual malaria transmission (transmission sustained by vectors that evade traditional control methods). Therefore, we propose refocusing surveillance efforts to ensure epidemiological relevancy by ensuring spatial and temporal overlap in epidemiological and entomological data, thus collecting mosquito data in concurrence with disease data.

There is presently a great push to invest in better surveillance, monitoring, and evaluation of malaria cases [5]. We believe this is an opportunity for mosquito surveillance to refocus in conjunction with disease surveillance, with more integration of both systems while sharing knowledge and resources. Côte d’Ivoire, Ghana, and Kenya are, for example, actively promoting an increased use of both epidemiological and entomological surveillance data for decision-making at all levels 33, 34, 35. We recommend that mosquito surveillance mirrors epidemiological surveillance both spatially and temporally. This is currently not the case. For example, in Burkina Faso, only 3 out of the 12 mosquito sentinel sites are matched with epidemiological surveillance. Similar disconnects are seen in Malawi and Zambia. A biological association in time and space of mosquito data and occurrence of clinical or subclinical infection (asymptomatic) in the human population is also needed. This includes a focus on highly anthropized (urban) and high travel areas (borders). Additionally, the importance of human dynamics should not be forgotten, as association in time and space of the mosquito species and local human population is important to understand disease transmission dynamics and focus vector control.

Mosquito surveillance is well-established for malaria yet limited for other mosquito-borne diseases such as arboviral diseases, while their outbreaks are becoming more common 6, 13••, 36, 37. Yellow fever vector surveillance in Ghana is limited to cross-sectional studies due to ad hoc funding, but processing *Aedes* collected during malaria surveillance, as suggested by the West African Aedes Surveillance Network [23], permits arbovirus surveillance to benefit from existing infrastructure. Only very few countries, such as Côte d’Ivoire and Cabo Verde, have arboviral vector surveillance systems in place 38, 39, which are both threatened by funding limitations. In the past, malaria vectors and other vectors were less likely to overlap in their host seeking behavior (malaria vectors at night indoors, arboviral vectors during the day outdoors). However, the dynamics are changing with increased overlap 31, 40, 41 and opportunities to combine surveillance activities. Malaria surveillance can complement other disease surveillance efforts, as sample handling and analysis are similar. Malaria surveillance staff can expand their focus to include arboviral diseases with minimal additional training. Initial steps can be taken with little additional resources by expanding malaria vector surveillance to the identification of all putative vectors and testing them for pathogens through xenomonitoring applications [42]. The tools currently available to collect mosquitoes are limited. They are largely unreliable outdoors, lack sensitivity to detect small changes, and do not collect information on vector behavior targeted by new tools such as the Attractive Targeted Sugar Baits and new bioactive agents (e.g. fungi or endosymbionts) 23•, 27, 43, 44•. We advocate for the development of novel mosquito collection tools that are sensitive, specific, and cost-effective [44]. Examples of novel tools include the electrocutting cage and small double-baited net trap [45]. Where possible, several collection methods should be used concurrently to capture a higher proportion of the vector population, including vectors of different diseases. Moreover, once samples are collected, we recommend all mosquitoes be identified to species morphologically and where indispensable (when identifying *An. gambiae* and *An. funestus* complexes) molecularly, with at least a proportion of samples investigated for parity and infective stage of pathogen by dissection, enzyme-linked immunosorbent assays (ELISA), or molecular tools, which are not standard practice in many surveillance systems. Additionally, the identification of blood meal sources using ELISA and molecular tools should be considered (especially for putative vectors) to demonstrate effective contact with humans.

## Increase the robustness of surveillance design

The successful control of malaria has resulted in more complex spatial and temporal variability [46] and the arboviral disease landscape remains largely unexplored. Yet, mosquito surveillance designs have changed little in the last decades, while technology has advanced greatly, providing new prospects. Surveillance sites are often chosen ad hoc, for example, based on accessibility, local relations, politics, and proximity to possible breeding sites. Furthermore, limited resources challenge the NMCP to select the most appropriate sentinel sites. Importance is given to the preservation of longitudinal time series. Evaluation on the relevancy and robustness of these sites is limited. Data from fixed sites collected at different frequencies by different stakeholders, though valuable, cannot always be generalized at a geographically relevant scale.

We recommend the re-evaluation of current surveillance sites in close collaboration with stakeholders, research centers, geospatial experts, and biostatisticians for a more spatially explicit sampling design to increase robustness and decrease biases. Sentinel sites are already being re-evaluated in Burkina Faso, Ghana, Malawi, and Zambia. The location of sentinel sites can be selected using geographical data on ecological niches and mosquito-borne disease transmission intensity (such as malaria) using open (satellite) data and standardizations [47]. Mosquito surveillance sites should also be informed by local human behavior, including parameters on sleeping routines and outdoor behavior, as it provides insight to times and locations of importance for disease transmission [48]. Sample size calculations can direct the optimum number of sites, replicates, and measuring frequencies to ensure objectives from the strategic plan are reached for informed decision-making processes. Tools are being developed to increase user-friendliness of these calculations, such as the Time and Space Sampling power tool (TIMESS) [49], which is a power analysis to estimate the number of locations and repeated measurement in an area with incorporation of seasonal patterns. The NMCPs can also employ adaptive surveillance designs that incorporate spatial and temporal aspects to adjust entomological sampling strategies upon availability of new information on ongoing transmission dynamics. Such epidemiologically relevant mosquito data can also be used to parameterize predictive models to deploy and improve interventions.

Global inequities in analytical capacity and limited funding for technician training and postgraduate training pose significant obstacles to effective mosquito surveillance 13••, 21••, 27. To overcome these challenges, we propose re-evaluating sentinel sites as a means to simultaneously train entomologists in open-access technological advances and utilizing opportunities for training and mentorship through regional organizations such as PAMCA (https://www.pamca.org), Vectopole Sud (https://www.vectopole-sud.fr/), vector atlas (https://vectoratlas.icipe.org/), and The African Center of Excellence in Biotechnological Innovations for the Elimination of Vector Borne Diseases (https://ace.aau.org/tag/cea-itech-mtv/).

## Conclusions

Weak mosquito surveillance systems hinder data-informed decision-making. Developing mosquito surveillance strategic plans with measurable outcomes and clear monitoring and evaluation systems are essential, even with limited resources. A centralized entomological data system should be developed and maintained with epidemiological relevance. Mosquito surveillance should mirror epidemiological surveillance, combining surveillance activities for multiple mosquito-borne diseases. Training as well as retaining talented and experienced entomologists is critical. Statistical and computational advances should be leveraged for more robust surveillance, with multidisciplinary support from social scientists, biostatisticians, epidemiologists, and local community members. Mosquito surveillance is increasingly seen as an intervention itself and requires the same rigor and focus.

## Funding

This work was supported by the 10.13039/501100000265Medical Research Council (MRC), United Kingdom [grant number MR/T031743/1 and MR/P027873/1], Wellcome Trust, United Kingdom [grant number 222019/Z/20/Z], and National Institute for Health and Care Research (NIHR), United Kingdom [grant number NIHR133144].

## CRediT authorship contribution statement

**Zanakoungo I**. **Coulibaly:** writing and reviewing, **Steve Gowelo**: writing and reviewing, **Issouf Traore**: writing and reviewing, **Rex B. Mbewe**: writing and reviewing, **Willy Ngulube**: writing and reviewing, **Evelyn A. Olanga**: writing and reviewing, **Adilson José dePina**: writing and reviewing, **Antoine Sanou**: writing and reviewing, **Sylvester Coleman**: writing, reviewing, and editing, **Julie-Anne A. Tangena**: conceptualization, writing, reviewing, and editing.

## Declaration of Competing Interest

The authors declare that they have no competing interests.

## Data Availability

No data were used for the research described in the article.
